# Knowledge, attitudes, and practices of healthcare professionals toward radiation safety in Kazakhstan: a cross-sectional study

**DOI:** 10.3389/frhs.2026.1779949

**Published:** 2026-03-11

**Authors:** Ainara Darbayeva, Tairkhan Dautov, Nurgali Nurmanbekov, Galiya Orazova, Aiman Musina, Elmira Yelshibayeva, Zarnigor Madumarova, Bakyt Duisenbayeva

**Affiliations:** 1 Astana Medical University, Astana, Kazakhstan; 2 National Scientific Medical Center, Astana, Kazakhstan; 3 Clinical and Academic Department of Radiology and Nuclear Medicine, University Medical Center, Astana, Kazakhstan; 4 Department of Inpatient Radiology, University Medical Center, Astana, Kazakhstan; 5 Department of Epidemiology and Biostatistics, Astana Medical University, Astana, Kazakhstan; 6 CT and MRI Department, RSE “Medical Centre Hospital of the President's Affairs Administration of the Republic of Kazakhstan”, Astana, Kazakhstan; 7 Department of Medical Radiology, Andijan State Medical Institute, Andijan, Uzbekistan; 8 Department Diagnostic Radiology, University Medical Center, Astana, Kazakhstan

**Keywords:** clinical decision-making, diagnostic imaging, ionizing radiation, Kazakhstan, radiation KAP

## Abstract

**Background:**

Clinicians’ overutilization of medical imaging increases patients’ exposure to ionizing radiation (IR). Assessing clinicians’ knowledge, decision-making, and potential for unnecessary imaging is therefore essential. This study aimed to investigate the knowledge, attitudes, and practices of healthcare professionals in Kazakhstan regarding imaging procedures involving IR.

**Methods:**

A cross-sectional questionnaire-based study was conducted between June 2024 and May 2025 to assess healthcare professionals’ knowledge, attitudes, and practices regarding medical imaging involving IR. A thirty-item questionnaire was used, including a knowledge section scored on a scale of 0–10, with higher scores indicating greater knowledge of ionizing radiation and imaging safety. Group comparisons were performed using independent *t*-tests or one-way ANOVA for continuous variables and chi-square tests for categorical variables. Multivariable linear regression was used to examine predictors of CT scan requests per month (treated as a continuous count variable), with a *p*-value ≤0.05 considered statistically significant.

**Results:**

Of 250 distributed questionnaires, 218 were completed (response rate: 87.2%). Nearly two-thirds (68.8%) of respondents were female, with a mean age of 29.5 ± 6.9 years, representing diverse medical specialties. The mean knowledge score was 50.5 ± 5.9, and did not differ by gender or geographic location (independent *t*-test, *p* > 0.05). However, clinicians aged >35 years and those working in public hospitals had significantly higher knowledge (one-way ANOVA, *p* = 0.010; independent *t*-test, *p* = 0.048). In multivariable linear regression, clinicians aged <34 years (*β* = 0.401; 95% CI: 0.202–0.300; *p* ≤ 0.05), those with <5 years of experience (*β* = 0.338; 95% CI: 0.311–0.403; *p* ≤ 0.05), and clinicians in high-volume urban settings (*β* = 0.455; 95% CI: −0.281 to 0.391; *p* ≤ 0.05) were associated with a higher number of CT requests per month. Notably, 24.6% reported ordering CT scans without clear clinical indications, influenced by workload pressures, patient expectations, fear of malpractice, and the need to expedite diagnostics. Additionally, 38% rarely reviewed prior imaging studies before ordering CT scans.

**Conclusion:**

There is an observed gap in clinicians’ knowledge and imaging practices. These findings highlight the need for targeted educational interventions, improved access to prior imaging records, and organizational strategies to optimize medical imaging practices and reduce unnecessary patient exposure to ionizing radiation. However, these results should be interpreted with caution due to the cross-sectional study design, convenience sampling, and reliance on self-reported data.

## Introduction

Exposure to low levels of ionizing radiation (IR) is a part of the natural environment. The estimated average annual radiation dose is around 3 mSv, with approximately 2.4 mSv originating from natural background sources such as radon and terrestrial radiation, and about 0.6 mSv attributed to anthropogenic sources, primarily medical diagnostic and therapeutic procedures (1). IR is a recognized carcinogen and poses a growing concern for patients undergoing diagnostic and therapeutic imaging worldwide.

IR is a known carcinogenic and presents a growing concern for patients undergoing diagnostic and therapeutic imaging procedures worldwide. In recent decades, the cumulative exposure to medical radiation has significantly increased, mainly due to the expanded use of computed tomography (CT), nuclear medicine, and interventional fluoroscopic techniques (2). This radiation increase has led to an increasing concern of potential health implications of radiation from medical imaging (3). Exposure to IR can induce molecular and genetic alterations, including DNA strand breaks, which may increase the lifetime risk of developing malignancies (4, 5). Importantly, the radiation doses delivered during standard CT examinations are comparable to those associated with an elevated cancer risk in exposed populations (6, 7). While an individual imaging procedure poses minimal risk, accumulating radiation doses through repetition, represents a significant public health concern. For instance, a single CT examination delivers a high radiation dose, and if repeated over time it may substantially increase the cumulative exposure (8). Moreover, unintended exposure to high levels of IR can cause acute tissue damage, manifesting as skin burns or temporary hair loss (9, 10).

Continuous technological innovation and the expanding use of imaging modalities, necessitate the need for effective safety regulations that become increasingly important to ensure responsible clinical application. Similarly, enhancing healthcare professionals' understanding of radiation protection (RP) through targeted education and regular training can substantially reduce unnecessary radiation exposure for both patients and medical personnel. A key strategy for reducing cumulative radiation exposure is to minimize medical imaging, which can be achieved by having a clear understanding of the decision making process.

In Kazakhstan, medical imaging utilization has increased rapidly over the past decade, with CT scanners concentrated primarily in urban tertiary hospitals (11). Referral pathways often depend on clinician judgment rather than standardized appropriateness criteria, and formal RP training is not universally mandated across specialties. Although national guidelines on radiation safety exist, implementation is variable and often limited by access to prior imaging records and limited awareness of cumulative radiation doses (12). These contextual factors distinguish Kazakhstan from other settings cited in the literature, where imaging protocols and RP training may be more standardized.

However, studies showed that clinicians often lack critical information such as patients' prior imaging records or cumulative radiation doses when requesting procedures involving ionizing radiation (9, 13). Another factor is the lack of awareness or sufficient understanding of established guidelines regarding the appropriateness of medical imaging for specific clinical situations, which might also lead to unnecessary imaging studies, resulting in avoidable radiation exposure for patients (13). Thus, the current study is a questionnaire-based cross sectional study aims to evaluate clinicians' knowledge, attitudes, and practices of requesting imaging procedures involving IR. Specifically, the study seeks to determine the level of knowledge of IR and RP principles among healthcare professionals in Kazakhstan. In addition, what attitudes and decision-making factors influence clinicians when requesting imaging procedures involving IR? As we as which clinician- or system-level factors (e.g., age, years of experience, workplace setting) are associated with higher frequency of CT scan requests? Hence, the study will enable the development of targeted educational policies to enhance radiation safety across various levels of clinical expertise.

## Methods

### Ethical approval

The study protocol reviewed and approved by the Local Bioethics Committee of Astana Medical University, Astana on 28/May/2024 (Approval No. 4). Prior to participation, all participants were provided with an explanation of the study objectives and procedures, and were asked to consent before proceeding with the study. Hence, informed consent was obtained from all individuals who agreed to take part in the survey.

### Sample size

The required sample size was estimated using a prevalence-based precision approach, appropriate for a descriptive cross-sectional study. Assuming a conservative prevalence of 50% (*p* = 0.50), a 95% confidence level (*Z* = 1.96), and a desired margin of error of ±7% (*E* = 0.07), the minimum sample size was calculated using the formula: *n* = (*Z*^2^ × *p* × (1 − *p*)) ÷ *E*^2^. Accounting for an anticipated 5% non-response rate, the total number of participants required was adjusted to 206, which is adequate to achieve reliable estimates of key indicators and feasible for a cross-sectional-based survey.

### The study design and setting

A 30-item questionnaire, adapted from Karavas et al. (14), aimed at assessing clinicians' awareness of radiation and the factors influencing their decisions to order imaging procedures involving IR. Specifically, items were updated to align with the latest International Commission on Radiological Protection (ICRP) recommendations and relevant guidance. Additionally, questions were reviewed to ensure consistency with Kazakhstan's national clinical imaging protocols and local regulatory requirements. The instrument divided into six domains: 1-assesses the demographics and professional information (age, gender, years of experience, specialty, and workplace setting). The second assesses self-perceived knowledge including clinicians' confidence in understanding radiation doses and safety principles. The third domain, investigates the objective knowledge of radiation, where 10 items included to assess the understanding of radiation exposure from common imaging procedures, e.g., CT, x-ray, fluoroscopy, as well as principles of radiation protection. The forth, CT request practices such as the frequency of CT scan requests within a typical month. The fith domain, investigates the patient interaction and prior imaging, for example, clinician perceptions of patient influence and access to prior imaging records. The sixth and final domain is decision factors and inappropriate imaging, such as factors influencing ordering of CT scans without clear clinical indication. An example of items included “Which imaging modality exposes the patient to the highest level of ionizing radiation?” and “How often do you review prior imaging studies before requesting a CT scan?”

The questionnaire initially developed in English and subsequently translated into Kazakh and Russian, the native and official languages in Kazakhstan, respectively. A standardized forward-backward translation process was used, and minor discrepancies in the Kazakh version were resolved through discussions between the research team and the translator. Content validity was assessed by a panel of five radiation safety and clinical imaging experts, who reviewed the translated instrument for relevance, clarity, and comprehensiveness. Items were revised as needed to ensure conceptual equivalence and clarity. The research team approved the final versions of the Kazakh and Russian translation and were subsequently used in the study.

### Sampling frame and data collection

Participants were recruited using a convenience sampling approach, targeting clinicians across multiple regions of Kazakhstan, including Astana, Almaty, and other urban and semi-urban healthcare institutions. Invitations were distributed via institutional email lists (*n* = 180) and professional social media channels, including WhatsApp and Telegram groups for medical professionals (*n* = 70), totaling 250 clinicians approached. To minimize duplicate responses, the online questionnaire platform was configured to allow one response per email address and IP address. No quotas or stratification were applied; the goal was to reach a diverse sample across specialties and geographic areas.

The questionnaire structured to capture multiple aspects of clinicians' practices and perceptions regarding radiation. The first section designed to collect demographic and professional information, including specialization, age, years of clinical experience, and professional rank. The second section aimed to assess the participants' self-perceived knowledge of radiation doses, and objectively evaluating their understanding of radiation exposure. The third section focused on clinician's practice of the frequency of CT scans requests within a typical month. The fourth section explored patients' inquiries and attitudes toward imaging procedures and how patients' prior imaging history influenced clinicians' decisions to order additional tests. The following part investigated factors that may affect clinicians' CT scan requests, while the final part of the questionnaire designed to analyze instances of CT imaging performed without clear clinical indication.

### Study validation and reproducibility

A pilot study was carried out to evaluate the validity and reliability of the questionnaire prior to its full implementation. This preliminary phase involved 25 healthcare professionals, representing the main professional groups targeted in the study. The primary objective was to assess the clarity, simplicity, and overall understandability of the survey items, ensuring that the language and terminology were appropriate for participants. Feedback from the pilot participants was carefully reviewed, leading to the revision and restructuring of several questions to improve clarity, remove ambiguities, and enhance the overall accuracy of the instrument. Data from this pilot phase were excluded from the main study analysis. The finalized version of the questionnaire was then used in the main study. However, the internal consistency of the questionnaire was evaluated prior to the main study. For the 10-item objective knowledge section, reliability was assessed using the Kuder-Richardson Formula 20 (KR-20), yielding a value of 0.72, which indicates acceptable internal consistency. For the multi-item Likert-scale domains, such as self-perceived knowledge, Cronbach's alpha was calculated and found to be 0.78, reflecting good internal consistency.

### Data storage

All materials including completed questionnaires and electronic records were securely stored with password protection and encryption to safeguard participants' privacy and maintain data integrity. Access to the research data was strictly restricted to the principal investigator, in full accordance with the ethical guidelines and data protection policies set by the institutional ethics committee.

### Statistical analysis

Data analysis conducted using IBM SPSS Statistics for Windows, version 25.0 (SPSS, Chicago, IL, USA). Descriptive statistics were used to summarize the participants' demographic characteristics. A multivariate analysis of variance (MANOVA) used to investigate the combined effect of demographic variables on multiple CT-related outcome measures. As the primary variables were independent categorical variables with nominal scales, the chi-square test was applied to compare frequency distributions. A *p*-value of less than 0.05 was considered statistically significant.

## Results

### Demographic data

A total of 250 questionnaires were distributed to participants using the Qualtrics online survey platform, of which 218 healthcare professionals from key regions across Kazakhstan completed the questionnaire, resulting in an overall response rate of approximately 87.2%. The majority of participants were females (68.8%), with an overall mean age of 29.5 ± 6.9 years, ranging from 25 to 56 years and an average of 5.0 ± 6.0 years of medical practice (range 1–21 years). This higher proportion of female respondents likely reflects the gender distribution among healthcare professionals in Kazakhstan, where nursing and certain medical specialties are predominantly female. Among them, 46.6% were specialists, 22.4% were general practitioners, and 31.2% others (including allied health). The majority of the respondents were from major government hospitals (41.3%), followed by private clinics (33%) and represented a variety of medical specialties, with internal medicine physicians comprising the largest group at 19.5% [Table 1].

**Table 1:** Demographic data of participants.

Variable	Number	Percentage
Gender		
Males	68	31.2
Females	150	68.8
Age group (years)		
25–29	92	42.2
30–34	65	29.8
35–39	30	13.8
40–44	18	8.3
45–49	9	4.1
50+	4	1.8
Work experience (years)		
1–5	101	46.3
6–10	67	30.7
11–15	32	14.7
16–20	13	6.0
21+	5	2.3
Workplace sector		
Government	90	41.3
Private	72	33
Others (e.g., small centers)	56	25.7
Urban	158	72.5
Rural	60	27.5
Department		
Internal medicine	43	19.5
General surgery	30	13.8
Orthopedic surgery	27	12.4
Cardiology	25	11.5
Pulmonary	22	10.1
Neurology	18	8.3
Somatology	15	6.9
Others	38	17.5
Position		
Specialist	101	46.6
General practitioner	49	22.4
Others (including allied health)	68	31.2

Total number = 218, Age rang = 25–56 years, and Mean age = 29.5 ± 6.9 years.

Mean number of years working=5.0 ± 6.0, and range 1–21 years.

### Knowledge analysis

The analysis of the association between gender and radiation knowledge showed no significant difference between male and female participants (mean difference: 1.1; 95% CI: −1.5 to 3.7; *p* = 0.42), nor across geographic regions or urban vs. rural institutions (mean difference: 0.5; 95% CI: −4.2 to 5.2; *p* = 0.31). Comparisons between two groups (e.g., gender, workplace sector) were performed using independent-samples *t*-tests, while comparisons across more than two groups (if applicable) were analyzed using one-way ANOVA. Assumptions of normality and homogeneity of variances were checked and reasonably satisfied; where assumptions were borderline, non-parametric Mann–Whitney *U* tests were performed as sensitivity analyses, yielding consistent results. Interestingly, clinicians older than 35 years demonstrated significantly higher knowledge scores (mean = 58.4 ± 8.8) compared to younger colleagues (mean = 41.7 ± 10.7), with a mean difference of 16.7 (95% CI: 9.8–23.6; *p* = 0.01). Additionally, participants working in public hospitals reported greater perceived knowledge (54.2 ± 5.5) compared to those in private institutions (47.0 ± 9.4), with a mean difference of 7.2 (95% CI: 0.2–14.2; *p* = 0.048) [Table 2].

**Table 2:** Association of demographic data and radiation protection knowledge scores.

variable	group	Mean knowledge score	*p*-value
Gender	Male	52.3 ± 8.1	0.42
	Female	53.4 ± 7.9	
Geographic region	Urban	53.0 ± 12.3	0.31
	Rural	52.5 ± 6.3	
Age group	≤34 years	41.7 ± 10.7	0.01
	≥35years	58.4 ± 8.8	
Workplace sector	Private hospital	47.0 ± 9.4	0.048
	Public hospital	54.2 ± 5.5	

### Multivariate analyses for monthly CT requests and demographic confounders

Multivariable linear regression models were used to examine associations between demographic characteristics and the frequency of CT scan requests per month [Table 3]. The outcome variable is the number of CT scan requests per month, and the *β* coefficients represent the estimated linear change in monthly CT requests associated with each predictor, holding all other covariates constant. All *β* coefficients are unstandardized, with units corresponding to CT scan requests per month. Covariates included simultaneously in the models (age, years of clinical experience, gender, and workplace setting) were selected *a priori* based on prior literature and theoretical relevance. Interaction terms, such as age or workplace sector, were explored but were not statistically significant and, therefore, not included in the final models. Model assumptions, including normality of residuals, homoscedasticity, and absence of multicollinearity were checked and satisfied. No significant association was observed between clinicians' gender and the number of CT requests per month (*β* = 0.601; 95% CI: −0.09 to 0.31; *p* = 0.41). In contrast, younger clinicians (<34 years; *β* = 0.401; 95% CI: 0.202–0.300; *p* ≤ 0.05), those with less than five years of clinical experience (*β* = 0.338; 95% CI: 0.311–0.403; *p* ≤ 0.05), and clinicians working in high-volume urban hospital settings (*β* = 0.455; 95% CI: −0.281 to 0.391; *p* ≤ 0.05) were associated with a higher number of CT requests per month (Table 3).

**Table 3:** Multivariate analysis of CT requests and demographic confounders.

Variable		Frequency of ordering CT scan per month	
		*β* (95% CI)	*P*-Value
Age	≤34 year olds vs. ≥35 year olds	0.338 (0.311, 0.403)	≤0.005
Gender	Male vs. Female	0.061 (−0.09, 0.31)	0.41
Years of service	≤5 years vs. >5 years	0.401 (0.202, 0.300)	≤0.005
Workplace sector	Private vs. public	0.455 (0.182, 0.391)	≤0.005

Note on Multiple Comparisons: Multiple demographic associations were explored. As these analyses were primarily exploratory, formal adjustment for multiple comparisons (e.g., controlling the false discovery rate) was not applied. *P*-values are interpreted cautiously, with emphasis on effect sizes and consistency across related outcomes rather than on statistical significance alone.

### Analysis of participants' attitude and practice

**Figure 1:**
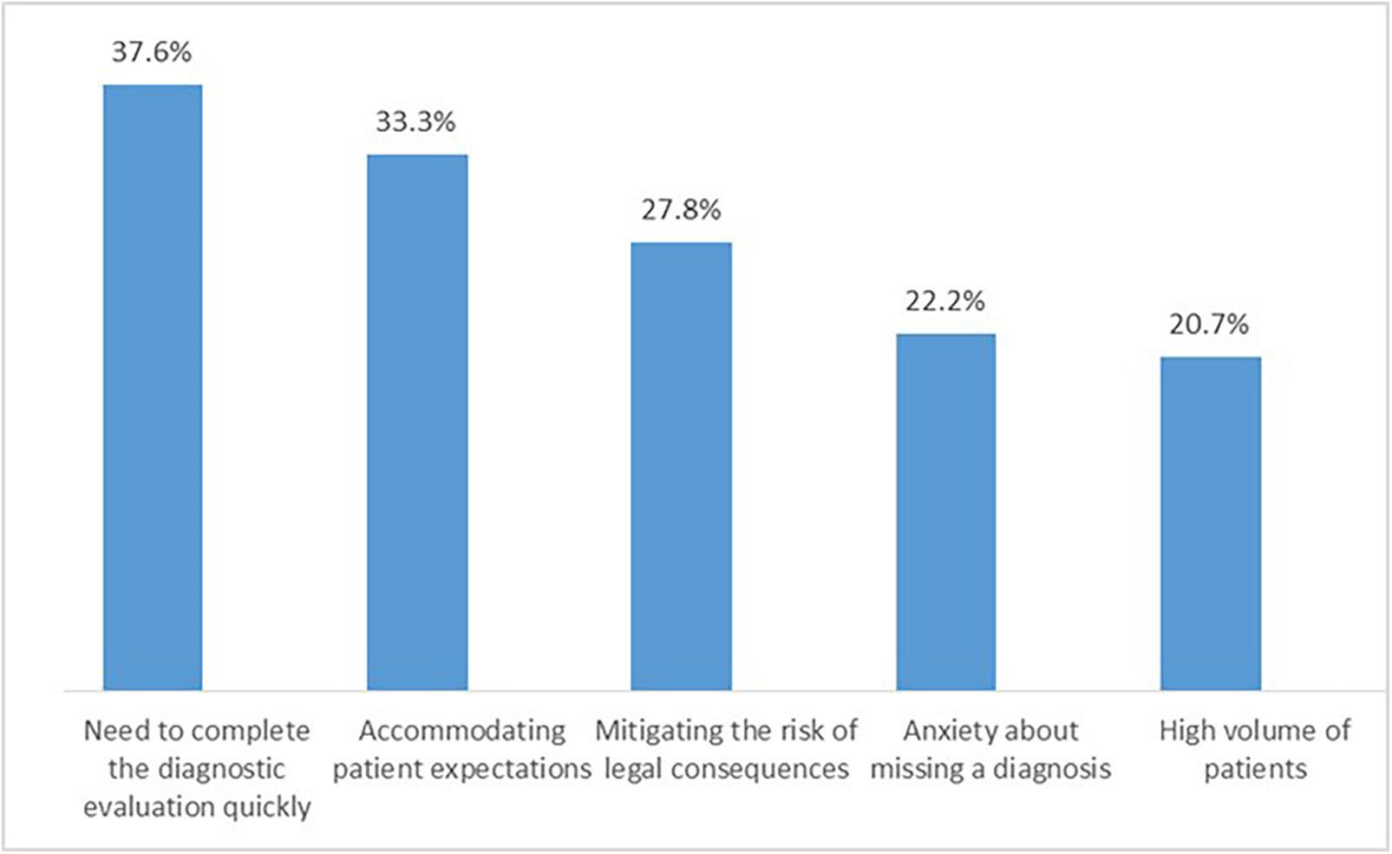
Reasons for ordering CT scans without clear clinical indications. Respondents were asked: “*Have you ever requested a CT scan when there was no clear clinical indication? If yes, what factors influenced your decision*?” Multiple answer options were provided: high patient volume, patient requests, fear of malpractice, concern about missing a diagnosis, and desire to expedite the diagnostic process. Respondents could select more than one factor. The figure shows the proportion of respondents selecting each factor.

Nearly one-quarter of respondents (24.6%) reported ordering CT scans despite the absence of a clear clinical indication (Figure 1). When asked about the reasons underlying this practice, participants identified multiple influencing factors, including patient requests, high patient volume, concern about potential malpractice claims, anxiety about missing a diagnosis, and the desire to accelerate the diagnostic process. The most frequently cited reasons were the need to complete the diagnostic evaluation quickly, followed by accommodating patient expectations and mitigating potential legal risks. These findings indicate that attitudinal factors, particularly, defensive medical practices, perceived diagnostic uncertainty, and responsiveness to patient demands, play an important role in imaging decision-making. Additionally, high workload and time pressure were widely perceived as contributors to increased CT utilization. Collectively, these results highlight that non-clinical attitudes and system-level pressures significantly influence CT ordering behavior and may contribute to imaging overuse. However, when asked about their willingness to participate in additional IR safety training and reduce the frequency of CT requests, the vast majority (78%) responded positively, indicating support for measures aimed at optimizing imaging practices. Furthermore, in response to questions about practical strategies to improve clinical practice, the most frequently cited measure reported by 67.2% of participants was to reduce patient workloads and provide clinicians with adequate time to perform thorough and careful examinations [Table 4]. Regarding the influence of patients' prior imaging history on clinicians' decisions, only a minority of respondents consistently reviewed previous imaging studies before ordering additional CT scans. Specifically, 32% reported that prior imaging moderately influenced their decisions, while 38% stated that they rarely reviewed prior studies, partly due to lack of interoperability between healthcare facilities, and incomplete electronic medical records. Combining these categories, only 30% of participants reported that they often or always review prior imaging, highlighting a gap in integrating past imaging history into clinical decision making.

**Table 4:** *Ad hoc* analysis of participants’ response.

Are you willing to participate in professional IR safety training that helps you reduce CT scan requests?		
Option	Number (n)	Percentage (%)
Extremely unwilling	5	2
Very unwilling	11	5
Moderately unwilling	11	5
Neither willing nor unwilling	22	10
Moderately willing	52	24
Very willing	58	27
Extremely willing	60	28
What is the most practical way to improve your clinical practice?		
Reduce patient workloads and provide clinicians with adequate time to perform thorough and careful examinations	147	67.2
Participate in continuing professional education and training programs	22	10
Implement clear clinical guidelines and protocols	15	7
Improve access to diagnostic tools and resources	14	6.4
Enhance teamwork and communication among clinical staff	10	4.6
Use decision-support tools or checklists to guide patient care	8	3.7
Other (please specify)	2	0.9
To what extent does a patient's prior imaging history influence your decision to order additional diagnostic tests (e.g., CT scans)?		
Not at all	8	17
Slightly important	18	39
Moderately important	32	70
Very important	25	55
Extremely important	17	37
Do you check a patient's prior imaging history before requesting a CT scan?		
Never	6	13
Rarely	38	83
Sometimes	27	59
Often	18	39
Always	11	24

## Discussion

The current study investigated the knowledge, attitudes, and practices of healthcare professionals toward imaging procedures involving IR in Kazakhstan. The results show that the healthcare professionals' understanding of IR varied significantly by age and practice setting. For example, physicians with more than five years work-experienced and those working in public hospitals demonstrated higher self-perceived and objective knowledge about IR compared to other participants. This finding aligns with earlier research that showed knowledge about radiation doses and health risks is often moderate to low among physicians. For instance, a systematic review reported that knowledge gaps in physicians' awareness of CT radiation dose and associated cancer risk differ between specialization and medical rank (15).

On the other hand, the observed higher knowledge scores among clinicians working in public hospitals and among older clinicians may reflect several factors, including selection effects, such as differences in who chose to respond to the survey, differential exposure to formal or continuing radiation safety training, or role-related differences in clinical responsibilities that influence familiarity with imaging procedures. Although stratified analyses or adjusted models could help explore these patterns further, the current cross-sectional design limits causal inference, and the findings should be interpreted as associations rather than evidence of direct effects.

However, in a study by Brown et al., that investigated the knowledge of medical imaging radiation dose and risk among healthcare professionals in Queensland, Australia, showed that only 17% correctly estimated the radiation dose from CT, and 10% of respondents mistakenly thought that CT radiation carries no increased lifetime cancer risk (16). Moreover, nearly a quarter of the participants (24.6%; 95% CI: 19.0%–30.5%) admitted to requesting CT scans in the absence of clinical indications. Nevertheless, this estimate was based on self-reported judgments by the respondents rather than an external evaluation against formal guideline criteria; therefore, it reflects participants' perception of appropriateness rather than an objective audit. These reasons included the need to complete the diagnostic process quickly, patient demand, and fear of malpractice. These reasons including the need to quickly complete the diagnostic process, patient demand, and fear of malpractice. These reasons mirror findings from other works indicating that workload pressures and patient expectations are significant determinants of imaging overuse (17). In a cross-sectional study by Karvas and colleagues that investigated doctor's level of awareness and reasons for requesting CT scans, showed that 23% of the surveyed professionals requested scans with no clinical indications, mostly due to workload, fear of malpractice, and patient demand (14).

This finding appears to be consistent with different previous research that reported many physicians, particularly in high-volume, emergency settings tend to order more imaging compared to others (18–20). The implications of overutilization of medical imagine are quite alarming. For instance, a recent modelling study by Smith Bindman et al., estimated that 93 million CT scans in the U.S. in 2023 could result in approximately 103,000 future cancers, estimated to be 5% of all new cancer diagnoses annually (6). However, these projections are based on U.S. utilization patterns and radiation doses, and may not directly apply to Kazakhstan, where CT use and population exposure may differ. The authors noted that, if current patterns of CT use and radiation dosing persist, cancers attributable to CT scans could eventually represent 5% of all new cancer diagnoses each year. While, non-clinical overuse is a well-documented practice, in a randomized controlled trial to investigate physicians' decision making in relation to medical imagine, Gimbel and colleagues suggested that physician decision can be influenced by safety and cost information and the order in which information is provided to physicians can affect their decisions (21). This finding emphasizes the dual nature of overutilization of medical imagine as not only due to poor knowledge, but also about behavior, system incentives, and risk perception (22–24).

However, participants identified high workload and time pressure as factors associated with more frequent CT scan requests, and the majority believed that reducing workload and patient density could help improve imaging practices and radiation safety. Allowing physicians more time for thorough clinical examinations by reducing patient loads may be associated with lower likelihood of requesting unnecessary CT scans and potentially reduce patients' exposure to IR (14). Interestingly, 78% of the participants expressed their willingness to engage in additional IR safety training, and ways to improve medical imagine practice. Education is likely to enhance clinical practice, as effective training demonstrated to raise clinicians' awareness of IR risks. For instance, a pilot pre and post intervention study by Young and colleagues showed that even brief educational interventions or a simple message about lifetime cancer risk from CT led to increased clinician awareness (25).

Overall, the results indicate that interventions to reduce inappropriate CT use should be multifaceted, including, educational efforts, and RP training, including dosage, risk, justification and optimization, which remain central in imaging-radiation safety frameworks (26). Similarly, access to previous imaging history and cumulative radiation exposure should be improved, since lack of such information was identified as a barrier to appropriate decision-making.

Beyond education, health services interventions could also support more appropriate imaging use. Clinical decision support (CDS) tools integrated with imaging order entry systems have been associated with improved appropriateness ratings of advanced imaging orders in some settings (27–29), although effects on overall inappropriate use have been mixed in others (30, 31). Similarly, studies of imaging referral guidelines and audit-and-feedback interventions suggest that systematic implementation can increase high-value imaging and decrease low-value referrals over time (32). Improving access to prior imaging history through integrated electronic records and embedding appropriateness criteria into routine workflows may also support better decision making. For Kazakhstan, a pragmatic implementation pathway could begin with pilot CDS and guideline dissemination in major hospital networks, linked to routine audit-and-feedback cycles, with impacts assessed in pre-post evaluations or cluster trials to determine effectiveness in local practice.

### Limitations

This study has several limitations. First, the cross-sectional design precludes establishing causal relationships between clinicians' knowledge, attitudes, and medical-imaging practices. Second, data were collected using a self-reported questionnaire, which may be subject to social desirability and recall bias; participants may have overestimated their knowledge or underreported inappropriate imaging requests. For instance, the reported 24.6% of clinicians ordering CT scans without clear indications reflects self-perceived appropriateness and was not verified against formal guideline criteria or hospital records, potentially underestimating the true prevalence of unnecessary imaging. Third, the study used a convenience sampling method, which may limit representativeness and reduce generalizability to all clinicians in Kazakhstan. This also introduces potential non-response bias, as clinicians with greater interest in radiation safety may have been more likely to participate, possibly inflating observed knowledge scores. Fourth, the study focused solely on clinicians' perspectives without including input from patients, administrators, or policy makers, which could provide a more comprehensive understanding of factors driving unnecessary imaging. Fifth, facility-level variables such as availability of imaging equipment, local protocols, or institutional guidelines were not captured; these unmeasured factors may confound associations between clinician characteristics and CT ordering patterns. Overall, these limitations may bias estimates toward overestimating knowledge and underestimating inappropriate imaging practices, and the magnitude of these biases is uncertain.

## Conclusion

In conclusion, this is the first study to examine clinicians' knowledge, attitudes, and practices towards IR and CT utilization in Kazakhstan, thus, addressing an important literature gap. This study highlights several key findings with practical implications. Younger clinicians and those with less experience demonstrated lower knowledge of radiation risks, suggesting a need for targeted educational programs and training to enhance awareness of safe imaging practices. Nearly one-quarter of respondents reported ordering CT scans without clear clinical indications, indicating that organizational and policy measures, such as decision-support tools, clearer guidelines, and workload management should be considered. Additionally, a number of clinicians rarely reviewed patients' prior imaging history before ordering additional tests, highlighting the importance of improving access to prior imaging records and promoting integration of past studies into clinical decision-making. Future research should assess the impact of these strategies using robust study designs, such as pre-post interventions, cluster randomized trials, or routine data audits, to determine their effectiveness in optimizing imaging practices and minimizing unnecessary exposure to ionizing radiation.

## Data Availability

The raw data supporting the conclusions of this article will be made available by the authors, without undue reservation.
